# Olfactory language and abstraction across cultures

**DOI:** 10.1098/rstb.2017.0139

**Published:** 2018-06-18

**Authors:** Asifa Majid, Niclas Burenhult, Marcus Stensmyr, Josje de Valk, Bill S. Hansson

**Affiliations:** 1 Centre for Language Studies, Radboud University, 6525 HT Nijmegen, The Netherlands; 2 Donders Institute for Brain, Cognition, and Behaviour, Radboud University, 6525HR Nijmegen, The Netherlands; 3 Language and Cognition Department, Max Planck Institute for Psycholinguistics, 6500 AH Nijmegen, The Netherlands; 4 Centre for Languages and Literature, Lund University, 22100 Lund, Sweden; 5 Humanities Lab, Lund University, 22100 Lund, Sweden; 6 Department of Biology, Lund University, 22362 Lund, Sweden; 7 Department of Evolutionary Neuroethology, Max Planck Institute for Chemical Ecology, 07745 Jena, Germany

**Keywords:** olfaction, culture, language, abstract, Jahai, Dutch

## Abstract

Olfaction presents a particularly interesting arena to explore abstraction in language. Like other abstract domains, such as time, odours can be difficult to conceptualize. An odour cannot be seen or held, it can be difficult to locate in space, and for most people odours are difficult to verbalize. On the other hand, odours give rise to primary sensory experiences. Every time we inhale we are using olfaction to make sense of our environment. We present new experimental data from 30 Jahai hunter-gatherers from the Malay Peninsula and 30 matched Dutch participants from the Netherlands in an odour naming experiment. Participants smelled monomolecular odorants and named odours while reaction times, odour descriptors and facial expressions were measured. We show that while Dutch speakers relied on concrete descriptors, i.e. they referred to odour sources (e.g. *smells like lemon*), the Jahai used abstract vocabulary to name the same odours (e.g. *musty*). Despite this differential linguistic categorization, analysis of facial expressions showed that the two groups, nevertheless, had the same initial emotional reactions to odours. Critically, these cross-cultural data present a challenge for how to think about abstraction in language.

This article is part of the theme issue ‘Varieties of abstract concepts: development, use and representation in the brain’.

## 1. Introduction

Odours give rise to primary sensory experiences. Every time we inhale we use our sense of smell to make sense of our environment. Odours are used to detect and identify foods, to avoid environmental hazards, and they enable social communication [1,2]. We can identify the source of a gas leak or navigate towards (or away from!) a department store perfume counter by using our sense of smell alone [3]. In fact, people can track a 10 m long scent trail of chocolate through a field [4]. Our ability to detect odours is so keen that the odorant ethyl mercaptan (which gives gas its distinctive smell) can be detected at concentrations equivalent to three drops diluted in an Olympic-size swimming pool [5].

Despite evidence of humans having such keen noses, it has been claimed that like other abstract domains—such as time or emotion—odours are difficult to conceptualize. We may be able to detect whether an odour is present or not, or follow it to its source using intensity gradients, but many doubt we can think olfactorily. The Scottish philosopher Thomas Reid claimed: ‘It is evidently ridiculous, to ascribe to it [the smell]^1^ figure, colour, extension, or any other quality of bodies. He cannot give it a place, any more than he can give a place to melancholy or joy: nor can he conceive it to have any existence but when it is smelled. So that it appears to be a simple and original affection or feeling of the mind, altogether inexplicable and unaccountable. It is indeed impossible that it can be any body: It is a sensation; and a sensation can only be in a sentient thing.’ [6].

Olfactory notions, indeed, seem difficult to conjure at will. When asked to imagine a pan of onions frying on a stove, only 3% of people say they are unable to see the onions, but a striking 57% say they are unable to smell them [7]. Similarly, when asked to imagine a specific object—for example, a rose—people rate the odour experience as having the lowest vividness and clarity in comparison to the other modalities [8,9]. Most striking, perhaps, is the finding that people rarely report olfactory imagery from their spontaneous conscious experiences: when participants are asked to report imagery they experience on the hour, every hour, visual images abound, occurring around 60% of the time; but olfactory imagery appears as little as 1% of the time [10]. This suggests that odour representations are fragile.

Similarly, odours appear difficult to verbalize [5,11–16]. Even when presented with familiar odours, speakers struggle to name them on the basis of smell alone [14–16]. When English speakers do name odours, they typically refer to their source (e.g. *smells like lemon*), suggesting that when they are able to identify an odour they think of it in concrete terms [17]. That is, odours trigger bounded, cohesive object concepts [18]. In fact, Hans Henning claimed: ‘olfactory abstraction is impossible. We can easily abstract the common shared colour—i.e. white—of jasmine, lily-of-the-valley, camphor and milk, but no man can similarly abstract a common odour by attending to what they have in common and setting aside their differences' [19, p. 66].

This view has been challenged recently by cross-cultural research. In some communities instead of talking about odours in terms of objects, dedicated vocabulary for referring to odour qualities is used instead [16,20–23]. These terms have been coined ‘abstract’ because they do not refer to any specific source. For example, in the language Jahai—spoken by a hunter-gatherer community in the rainforest of the Malay Peninsula—the term *cŋɛs* is used to refer to stinging sorts of smells associated with petrol, smoke, and various insects and plants, while *pl?ʔeʕŋ* refers to bloody, fishy, meaty sorts of smells. There are around a dozen smell terms in Jahai: they are monolexemic, stative verbs; they do not refer to a specific or restricted class of objects; they are also psychologically salient, appearing in everyday conversation and child speech [16,20]. Under experimental conditions, Jahai speakers depart from English speakers in how they encode odours too. Majid & Burenhult [16] gave 10 male Jahai participants and 10 age- and gender-matched English participants common odours (familiar to Westerners, e.g. chocolate, petrol) and asked speakers to name them. They found that English speakers indeed used source-based, concrete vocabulary, while Jahai speakers used abstract odour terms. In addition, English participants had very low agreement in how they described odours, whereas Jahai participants had significantly higher agreement.

At the same time, it has been suggested that the main evolutionary function of the olfactory system is to detect odour pleasantness, so that people universally perceive the same odours as pleasant versus unpleasant on the basis of the physical structure of the odour molecule [24–26]. This raises a puzzle because in a separate line of inquiry researchers have proposed that abstract concepts are more detached from sensory experience than concrete concepts, and more variable cross-linguistically [27,28]. If Westerners truly think about odours in terms of concrete objects, whereas some small-scale communities think about odours as abstract qualities, might there be knock-on consequences for their underlying odour concepts? Consistent with this proposal, cross-modal associations between odours and colours have been shown to differ as a function of how the odour is described: odours described using source-based terms give rise to more consistent and canonical colour associations (e.g. banana odour → ‘*smells like banana*’ → colour yellow) than those described with abstract terms (e.g. *musty*), which instead have less strong colour associations [17]. This suggests abstract odour vocabulary is less grounded in multi-modal sensory experience. Accordingly, this predicts that Jahai participants who describe odours with abstract vocabulary might have weaker emotional associations to odours. Conversely, abstract words are said to be more emotionally-loaded [29,30], which would predict that Jahai participants should evince stronger emotional reactions to odours as a function of their specific linguistic encoding.

The current paper re-visits the issue of olfactory abstraction across cultures, and investigates its interaction with emotion. We asked a larger sample of men and women in Jahai and Dutch to name monomolecular odorants, while measuring both verbal (odour names and reaction times) and non-verbal (facial expression) responses. We used ‘abstract’ monomolecular odorants (not clearly associated with any concrete source) to investigate whether this would lure Western participants to produce more ‘abstract’ verbal responses; thus testing task parameters for abstraction in odour naming [31]. In addition, we sought to establish whether Jahai and Dutch participants had ‘universal’ non-verbal responses to odorants or whether emotional reactions varied cross-linguistically. That is, do Jahai and Dutch participants find the same odours pleasant and unpleasant (as measured by their facial expressions), but only later differentiate in terms of their verbal responses; or do the two groups differ in their initial, affective reactions to odours too?

## 2. Methods

### (a) Participants

Participants were 30 (15 women) native speakers of Jahai. Age could only be estimated for most people, but ranged from 15 to 64 years; approx. *M* = 32 years. Thirty Dutch participants were matched to Jahai for age and gender; with equal numbers of men and women, age *M* = 32 years (range 16–64). Jahai and Dutch did not differ by age *t*(58) = 0.18, *p* = 0.99. All Jahai still pursue traditional foraging, although they reside in a resettlement village much of the time, and so are exposed to modernity. They were tested in Air Banun, Hulu Perak district, Peninsular Malaysia. The Dutch participants live a typical urbanized Western lifestyle. They were tested in Nijmegen, The Netherlands. Although participants were matched as closely as possible, there were nevertheless substantial differences between the groups, including in schooling and multilingualism. Seventeen Jahai participants had no schooling, and the rest had only 1–6 years of primary school education (in Malay). Most Jahai speak Malay, and many are also fluent in Temiar (a related Aslian language). All Dutch participants were educated to at least high school level; 10 participants also had university-level education. All (but one) Dutch participants were also fluent in English, and many spoke at least two additional languages (including German, French, Greek, Chinese, etc.). All Jahai men, and more than half the women smoked; only eight Dutch men and two Dutch women reported they smoked.

### (b) Stimuli

Since most commercially available smell tests are primarily comprised of odours of pleasant fruity and flowery notes, we devised a novel set of test odours constructed to better match what we knew about the odour words used by the Jahai. These included odours for more varied unpleasant smells. Specifically, we selected a set of monomolecular odours (*n* = 37),^2^ which apart from fruity and flowery smells, also included volatiles associated with, e.g. animals, decay, meat, blood, mould and faeces (table 1). The odorants were soaked into the cotton filament of a plastic marker pen ‘Sniffin' Stick’; the cap was removed by an experimenter during trials, and the felt-tip placed in front of participants' nostrils.

**Table 1.** Odorants with their unique numerical identifier assigned by the Chemical Abstracts Service (CAS), with their associated chemical name and brief descriptors.

number	CAS#	name	descriptor
2	513-86-0	acetoin	butter, cream
4	142-62-1	hexanoic acid	fatty, fruity
6	79-09-4	nonanoic acid	cheesy, pungent
8	137-00-8	sulforol	meaty, beefy
9	75-50-3	trimethyl amine	fishy
12	5655-61-8	bornyl acetate	balsamic, woody
13	80-71-7	cyclotene hydrate	caramel
15	104-67-6	gamma-undecalactone	peachy fruity
18	290-37-9	pyrazine	nutty
20	87-44-5	beta-caryophyllene	spicy, clove
21	123-25-1	diethyl succinate	fruity
22	123-32-0	2,5-dimethyl pyrazine	chocolatey
23	105-54-4	ethyl butyrate	pineapple, fruity
24	55704-78-4	meaty dithiane	meaty
25	135-79-5	isopropyl quinoline	green
27	1222-05-5	musk gx 100%	musk
29	16409-46-4	menthyl isovalerate	green woody sweet
32	78-96-6	1-amino-2-propanol	fishy
33	71-41-0	amyl alcohol	winey, yeasty, fermented
34	83-34-1	skatole	faecal
35	96-15-1	2-methyl butyl amine	fishy
36	103-09-3	isooctyl acetate	earthy
37	109-05-7	2-methyl piperidine	fishy
39	1878-18-8	2-methyl-1-butane thiol	bloody, sulphurous
40	4861-58-9	2-pentyl thiophene	bloody (fruity?)
41	18138-04-0	2,3-diethyl-5-methylpyrazine	musty, nutty, hazelnut
44	59558-23-5	para-cresyl caprylate	faecal
45	99-87-6	para-cymene	terpenic, rancid, woody, citrus, spicy
46	3391-86-4	1-octen-3-ol	earthy, mushroom
47	45019-28-1	4-methyl nonanoic acid	meaty
49	5333-83-5	1-(2-thienyl) butanone	grilled meat
51	625-33-2	3-penten-2-one	fishy, fruity?
53	80-56-8	alpha-pinene	piney
54	3681-71-8	z3 hexenyl acetate	sharp fruity-green
55	106-25-2	nerol	sweet, floral, rose
56	122-03-2	cuminaldehyde	green, herbal, spicy, characteristic cumin
57	76-22-2	camphor	camphoraceous

### (c) Procedure

Participants were tested in their native language, i.e. Jahai and Dutch. There were two experimenters: one who operated a camera directly opposite the participant; and a second who presented the participant with the odorants, who sat at 90° to the participant. Odours were presented one at a time with at least 60 s between different odorants. Participants were told to keep their head still while the experimenter brought the Sniffin' Stick to their nose so they could smell it, and then said ‘What smell is this?’: in Jahai 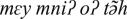 (literally, what smell 3S DEM ‘what smell it this’); in Dutch *Welke geur is dit?* (literally, ‘what smell is this?’). The participant could request to smell an odour again as many times as they wished, and were frequently asked if they would like to take a longer break. The whole session took approximately 1 h to run per person.

### (d) Coding

After testing, recordings were imported into ELAN [32]. ELAN enables annotation of both verbal and non-verbal responses with high timing accuracy in a single platform. The full verbal responses to each odorant were transcribed, and main contentful responses coded. For example, from the full Dutch response: *Ja, het heeft iets kaneligs maar ik kan, nee, ik kan, eh, niet specifiek, eh, zeggen wat het is.* ‘Yes, it is something cinnamon-like, but I can, no, I can, erm, not specifically, erm, say what it is.’; ‘cinnamon’ was coded as the main response, which is a source-based description. Onset and offset of transcriptions were time-aligned with speech. Time to name an odour (in ms) was measured from when the Sniffin' Stick was closest to the nose to when the participant began to speak. Ideally exact sniff measurements would have been taken but this was not practical in the field, and more critically would have disrupted facial expression coding. Separately, a trained FACS coder (Facial Action Coding System [33]) annotated the non-verbal facial responses to each odour. For the current purposes, we focus on facial muscle movements (action units, AU), previously identified as being relevant for ‘disgust’ or ‘pleasure’ [34,35], or which were recurrent in the dataset (table 1). Our focus was on the first appraisals, and so only those AUs that began within 2 s of the Sniffin' Stick being held to the nose were coded.

Once transcribed and coded, data were extracted from ELAN and the following dependent variables were examined: (i) type of responses given to odorants; (ii) length of descriptions to odorants for Jahai and Dutch; (iii) timing of responses—i.e. how long participants took to give a verbal response measured from when the Sniffin' Stick was held at the nose; (iv) agreement in descriptions across communities; and (v) facial expressions to odours.

## 3. Results

### (a) Type of response

**Figure 1.**
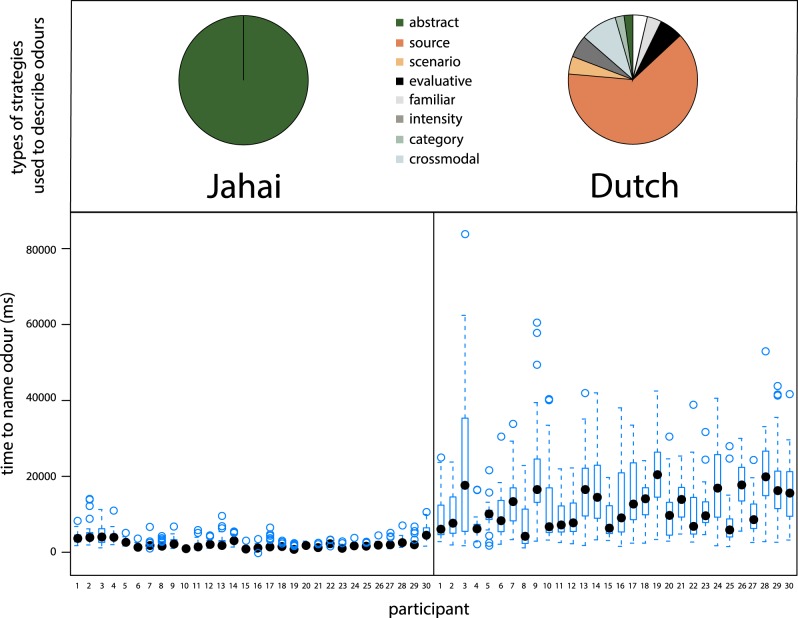
(Top panel) Types of strategies used to describe odours by Jahai and Dutch participants, and (bottom panel) time taken to name odours by language and participant. Jahai speakers use overwhelmingly abstract odour terms and take around 2 s to name odours; Dutch participants use predominantly concrete descriptors and take around 13 s to name odours.

There was a clear qualitative distinction between Jahai and Dutch responses (figure 1). Out of 1110 opportunities to describe odours (37 stimulus × 30 participants), Jahai participants produced 22 distinct response types: 19 were abstract smell terms, and these made up 99.5% of all tokens. In addition, there were 3 source-based terms (referring to types of plants; 0.3%); 1 evaluative expression (be good; 0.1%); and only once did 1 participant produce no verbal descriptor (0.1%).

In contrast, Dutch participants produced 707 distinct responses, and more diverse strategies. Participants said they didn't know what the odour was 3.7% of the time, and were able only to say that it was familiar in some way 3.9% of the time; or they produced an evaluative response 5.7% of the time (e.g. *wow, godver* ‘damn’, *gek* ‘crazy’). The dominant contentful response was to refer to a concrete source (e.g. *bloemen* ‘flowers’; *ammoniak* ‘ammonia’, *mest* ‘manure’, etc.); this category consisted of 557 different tokens and 64.7% of all responses. Another strategy was to refer to a specific scenario (4.7%) (e.g. *als je d'r langsfietst of achter de vuilniswagen staat niet d'r bovenop* ‘if you ride along or stand behind a garbage truck, but not right on top of it’; *eten dat lekker smaakt maar als het in de pot bij oma ligt dan stinkt het* ‘food that tastes good but if it is in the pot at granny's then it stinks’; *huis waar niet veel gelucht wordt* ‘house that isn't aired’). Thereafter, responses could be said to involve some degree of abstraction: for example, participants used a crossmodal metaphor of some type 9.2% of the time (e.g. *scherp* ‘sharp’, *zoet* ‘sweet’, *warm* ‘warm’); or gave a generic category for some odours (2.3%) (e.g. *chemisch* ‘chemical’, *synthetisch* ‘synthetic’, *natuurlijk* ‘natural’, *organisch* ‘organic’), in which they referred to a type of odours; or they referred not the quality of the smell but its intensity (5.7% of responses). There were only five abstract odour terms used throughout the study (*stinkt* ‘smelly’; *stinkt niet* ‘not smelly’; *muf* ‘musty’; *ranzig* ‘rancid smell’; and *weeïg* ‘sickly smell’) and these made up only 2.2% of all responses.

### (b) Length of response

Further confirming the qualitative differences between Jahai and Dutch verbal responses, we found that Jahai speakers gave a single abstract term the majority of the time, and as such the average length of their response was much shorter than Dutch responses. Prior research has established that orthographic length correlates highly with phonetic length, even for languages with irregular spelling [36]; as such we took orthographic length as a proxy for speech length. Arguably a strictly phonemic representation of Dutch responses, comparable to the Jahai orthography [37], would result in somewhat shorter estimates than those measured here, but the differences between languages were nevertheless substantial: Jahai responses were on average five characters long, whereas Dutch responses were 85 characters. Using the lme4 package [38,39], linear mixed-effects models were fitted to the log-transformed data (which were otherwise skewed). Language was treated as a fixed effect, with participants and items as random effects; *p*-values were obtained by likelihood ratio tests of the model with and without language as a factor. We found that language had a significant effect on the length of responses (measured in characters) *χ^2^*(1) = 3660, *p* < 0.0001.

### (c) Time of response

Not only were the Jahai more succinct in naming odours, they were quicker too: on average Jahai participants took around 2 s to give a verbal response, whereas Dutch participants took more than 13 s (figure 1). Dutch participants took even longer if time to produce a contentful response was measured (e.g. ‘flowers’; as opposed to saying ‘I don't know’ or equivalent). Linear mixed-effects models were fitted (as above) to the log-transformed time (in ms) that it took participants to name each odour. Four datapoints were clearly outliers based on visual inspection of the data (0.18%) and were removed from both analyses reported below. Language had a significant effect on time to produce a first verbal response *χ^2^*(1) = 2411, *p* < 0.0001, and first contentful response *χ^2^*(1) = 2689, *p* < 0.0001. In fact, to produce the first contentful response, Jahai speakers took *M* = 2727 ms, whereas Dutch speakers took 17 280 ms.

### (d) Agreement

Jahai participants agreed more with one another in how to describe each stimulus than Dutch speakers did. We calculated agreement across speakers in naming each odorant separately for Jahai and Dutch using Simpson's Diversity Index [40], following [16]. We asked whether despite differences in linguistic strategies, there was nevertheless consensus when odours were described. Language had a significant effect on agreement calculated over first responses *χ^2^*(1) = 122, *p* < 0.0001, and when taking all responses into consideration *χ^2^*(1) = 131, *p* < 0.0001. That is, Jahai participants agreed more with each other in how to describe odours, even when all responses Dutch participants gave were considered in comparison.

**Figure 2.**
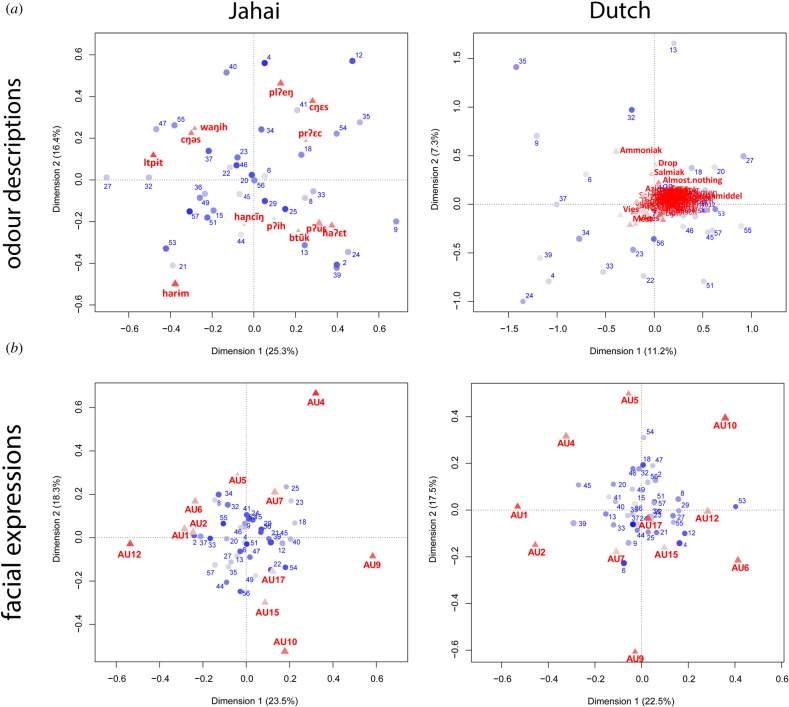
Correspondence analysis plots of (*a*) odour descriptions (red) and odorants (blue) for Jahai and Dutch and (*b*) facial expression action units (AUs) by odorants. See table 1 for full description of odorants and table 2 for description of AUs.

Next, we examined the data using a dual-factoring technique—correspondence analysis (ca package in R [41])—which enables visualization of associations between categorical variables. Figure 2 (*a*) depicts the relationship between verbal labels^3^ (red) and odorants (blue; see table 1 for list of odorants). In these plots, the more two odorants are called by the same term, the closer they are plotted together. Similarly, the more often terms are used for similar odorants, the closer they are plotted. The Dutch data showed weak structure, as reflected in the poor model fit (only 18.5% of the variance in the data are captured by the first two dimensions).^4^ Verbal responses mostly cluster on 0, suggesting weak associations between specific labels and particular odorants. Odour stimuli were, nevertheless, dispersed because they were rarely called by the same term.

**Table 2.** Action units (AUs) coded for facial expressions, their brief description, and correlation values (Pearson *r*) across odorants between Jahai and Dutch participants (with *p* one-tailed; *df* = 35).

action unit	description	*r*	*p*
AUs associated with pleasant emotions			
AU1	inner brow raise	0.033	0.423
AU2	outer brow raise	−0.087	0.305
AU6	cheek raise	0.295	0.038
AU12	lip corner pull	0.360	0.014
AU17	chin raise	0.234	0.082
AUs associated with unpleasant emotions			
AU4	brow lower	0.461	0.002
AU7	lid tight	0.520	0.000
AU9	nose wrinkle	0.292	0.040
AU10	upper lip raise	0.290	0.041
AU15	lip corner depress	0.105	0.268
AU5	upper lid raise	−0.045	0.396

The Jahai name∼odorant plot looks strikingly different (figure 2*a*). The first two dimensions explained 41.7% of the variance. There was a clearer correspondence between labels and odorants. The left-hand side depicts terms that prototypically refer to pleasant odours, while the right features terms prototypically referring to unpleasant odours (interpreted according to the semantics of the Jahai terms [20]). Correspondingly, odorants (blue) on the left are classified by Jahai terms as more pleasant than those on the right. So, musk (27), nerol (described in the literature as sweet, floral; 55), alpha-pinene (piney; 53), diethyl succinate (fruity; 21), champher (camphoreous; 57); 3-penten-2-one gamma-undecalactone (peachy fruity; 15), etc. (described in the literature as both fishy and fruity; 51) are all described with terms for fragrant-type odour words. In the same space we also find 4-methyl nonanoic acid (meaty; 47), 1-amino-2-propanol (fishy; 32), 2-methyl piperidine (fishy; 37), which are described with these general fragrant terms, and specifically *cŋəs*, which could be glossed as ‘edibly fragrant’. Note, however, that other odorants that have previously been described in the olfactory literature as ‘fishy’ and ‘meaty’ load on the other side of the plot, and are described by the Jahai with terms best glossed as ‘stinking’; e.g. *haʔɛ̃t*: trimethyl amine (9), meaty dithiane (24), etc. This is likely due to the subtle differences in the odour characteristics; e.g. trimethyl amine is said to be rancid and sweaty, as well as fishy in odour.

### (e) Emotion

From the ELAN coding of facial expressions, we identified whether each participant displayed the target AU (table 2) for each odorant. We then aggregated Jahai (*n* = 30) and Dutch (*n* = 30) responses for each AU separately, and examined whether the two groups displayed similar emotional reactions to odorants, or not. To test this, we correlated summed AUs displayed by each group: if Jahai and Dutch participants have similar responses to odorants, then facial expressions ought to covary. Table 2 shows that AUs associated with negative facial expressions [34,35] for odorants correlated for the Jahai and Dutch (as reflected in brow lower, lid tight, nose wrinkle and upper lip raise); as did positive facial expressions (i.e. cheek raise and lip corner pull), albeit less clearly (see also figure 2). This suggests that despite differential linguistic categorization of odours, the two groups nevertheless converge on their initial affective responses.

## 4. Conclusion

Olfactory abstraction varies across cultures: while Dutch participants confirmed the often-touted claim that ‘olfactory abstraction is impossible’ [19] by providing mostly concrete language in response to odours, Jahai speakers overwhelmingly described odours with dedicated, abstract language. In addition, their responses were faster and shorter, providing converging evidence that the Jahai are communicatively adept in talking about odours.

Even for the monomolecular odours used in this study, which do not have a single object entity associated with them, Dutch participants predominantly tried to identify a source (e.g. flowers), or situation (e.g. house that isn't aired), corresponding to that aroma. Their grappling to identify concrete sources was in sharp contrast to the fluent abstract Jahai responses. Previous studies have shown that Standard Average Europeans struggle to identify odours [5,13–16], as also illustrated by the Dutch here. The greater ease of linguistic expression demonstrated by the Jahai is not unique, however. It appears that hunter-gatherer communities in particular find odours easier to talk about [23].

Despite these differences in language, both groups appeared to have similar initial affective responses to odours—as measured by facial expressions—consistent with previous proposals of universally pleasant odours [24–26]. This is not an obvious result, as others have suggested that abstract concepts are more detached from sensory experience [27,28], or conversely that they are particularly valenced [29,30]. As such, we could have expected the groups to diverge in their emotional expressions, so that their facial expressions were in line with their verbal content. We did not find compelling evidence of this. Note, however, that the groups could have diverged later in their facial expressions when they had fully lexicalized their conceptual content. We did not directly asses this. Instead, we focused on initial facial expressions that began within the first 2 s of participants sniffing an odour, and found strong similarities in this time window.

Facial expressions are, of course, dynamic. In our data, after first appraisal, facial expressions often reflected ‘thinking’ expressions, perhaps associated with trying to retrieve words. Thereafter, participants often showed signs of ‘positive’ emotion: they frequently laughed—particularly after perceiving unpleasant smells. This laughter arguably reflects a response to the social situation of being asked to smell ‘disgusting’ smells. Consistent with this, the laughter was often accompanied by direct eye-gaze with the experimenter. Given the large differences in time to verbalize odours by Jahai and Dutch participants, it is unclear whether facial expressions at time of verbalization would provide reliable comparative data of emotion to the odour *per se*.

The fact that both groups display similar emotional reactions to odours initially, but later diverged in their linguistic encoding thereafter provides fresh perspective on the controversial issue of whether olfactory perception proceeds by determining valence first [5,24] or by identifying a bounded, cohesive odour concept foremost [18]. Contrary to previous reaction time studies [42,43], we find that behaviourally relevant responses strongly support the valence-first theory: within the first 2 s the face already communicates whether an odour is positive or negative, but verbal identification does not happen until around 2.7 s for the Jahai, and takes almost 17 s for Dutch speakers.

To conclude, odours may initially be treated in similar ways according to their pleasantness across diverse communities. But the fact that they vary in their linguistic expression across cultures suggests that the notion of what is ‘abstract’ or ‘concrete’ is in part a culturally-contingent fact.

## Endnotes

1 Material in brackets added; not in the original text.

2 Jahai participants also responded to an additional seven odours that we do not include here.

3 Only terms that appeared in the database with a frequency greater than 5 were considered in these analyses to ensure model convergence.

4 Based on the scree-plot, the next cut-off would be at five dimensions, which would then cumulatively account for 37.9% of the data.

## Data accessibility

The data are accessible from The Language Archive https://hdl.handle.net/1839/1d183755-be22-4d55-afc8-7a691b0f09bd.
